# Review: *Salmonella* Dublin in dairy cattle

**DOI:** 10.3389/fvets.2023.1331767

**Published:** 2024-01-09

**Authors:** Ana Velasquez-Munoz, Rafael Castro-Vargas, Faith M. Cullens-Nobis, Rinosh Mani, Angel Abuelo

**Affiliations:** ^1^Department of Large Animal Clinical Sciences, College of Veterinary Medicine, Michigan State University, East Lansing, MI, United States; ^2^Departamento de Ciencias Veterinarias y Salud Pública, Universidad Católica de Temuco, Temuco, Chile; ^3^Agriculture and Agribusiness Institute, Michigan State University Extension, Michigan State University, East Lansing, MI, United States; ^4^Veterinary Diagnostic Laboratory, College of Veterinary Medicine, Michigan State University, East Lansing, MI, United States

**Keywords:** latent carrier, risk factors, prevention, calf health, zoonosis

## Abstract

*Salmonella enterica* serovar Dublin (*S*. Dublin) is a bacterium host-adapted to cattle with increasing prevalence in dairy facilities. It can severely affect cattle health, producing high morbidity and mortality in young calves and reducing the performance of mature animals. *Salmonella* Dublin is difficult to control and eradicate from herds, as it can be shed from clinically normal animals. In addition, *S*. Dublin is a zoonotic bacterium that can be lethal for humans and pose a risk for human and animal health due to its multi-drug resistant characteristics. This review provides an overview of *S*. Dublin as a pathogen in dairy facilities, the risk factors associated with infection, and current strategies for preventing and controlling this disease. Furthermore, current gaps in knowledge are also discussed.

## 1 Introduction

*Salmonella enterica* subspecies *enterica* serovar Dublin (*S*. Dublin) is a Gram-negative bacterium commonly affecting dairy cattle. *Salmonella* Dublin is host-adapted to cattle, where it can cause severe disease and compromise the welfare of young and mature bovine, and the economic return of the producer (1–4). Moreover, *S*. Dublin is a zoonosis that can cause severe disease leading to hospitalizations and mortality of consumers, farm personnel, calf handlers, veterinarians, and their families (5, 6). In France, major outbreaks had occurred as a result of the consumption of raw bovine cheese (7). Some countries like Denmark initiated a surveillance and control program since 2002, and as a result, the prevalence of *S*. Dublin in cattle was reduced from 25 to 7% from 2002 to 2015 (8). In countries without a control program, however, the prevalence of infections is high. For example, reports from Great Britain and Canada document apparent prevalence of infected herds ≥25% (9, 10). Also, *S*. Dublin has been the most frequently identified serotype among bovine *Salmonella* isolates from clinical samples submitted to veterinary diagnostic laboratories in the US and UK (11–14), and the second most common serovar in registered salmonellosis outbreaks in Germany, accounting for 30%−40% of cases (15). In the European Union, Dublin was also the second most common *Salmonella* serovar in cattle following Typhimurium (16).

In the US, *S*. Dublin has become one of the most important multi-drug resistant (MDR) bacteria in cattle (5, 17). The MDR has complicated the treatment of clinically sick animals and has become a threat to human medicine (18). In addition, *S*. Dublin may be difficult to control and eradicate from positive herds, as infection may persist in latent carriers and intermittently be shed to the environment (2). For the reasons provided, this review will focus primarily on risk factors for infection, prevention, and control of *S*. Dublin in dairy herds.

## 2 *Salmonella* Dublin in animal and human health

### 2.1 Prevalence in dairy farms

*Salmonella* Dublin is present worldwide but estimates of the proportion of *S*. Dublin infected herds vary greatly by country (Table 1). Some European countries have established a *S*. Dublin control and eradication that includes routine testing of all farms (19, 25, 26). Although no country is free from salmonellosis, 9 European countries report only sporadic cases. For example, *S*. Dublin has not been detected in Finland since early reports in the 1980s (26). Some countries, namely Finland, Norway, and Sweden, have additional restrictions for cattle trade in place (27). Conversely, more limited information regarding the prevalence of *S*. Dublin is available in countries without control programs. However, *S*. Dublin has been identified as one of the most common isolates of *Salmonella* spp. in dairy farms in the US, Germany, and the UK (11–15). In 2014, the USDA's National Animal Health Monitoring System (NAHMS) conducted a cross-sectional study including 234 farms nationwide. *Salmonella* Dublin was present in 0.7%, 6.7%, and 1.8% of the operations, milk samples, and milk filters, respectively (23). Additionally, the University of Minnesota Veterinary Diagnostic Laboratory (VDL) determined that *S*. Dublin was the most prevalent serotype isolated from bovine samples between 2005 and 2014, representing 31.8% of all isolates examined from 880 dairy farms from the upper Midwest (11). Likewise, *S*. Dublin was the most prevalent serotype in bovine samples in the University of Wisconsin VDL, accounting for 23% of all isolates from 2006 to 2015 (12). Similarly, *S*. Dublin has been the most common *Salmonella* serovar isolated from bovine samples at the Michigan State University VDL between 2018 and 2022, representing 10%−20% of all bovine *Salmonella* isolations (Table 2). In Germany and Italy, however, *Salmonella* Typhimurium was the most frequently isolated serovar in cattle samples collected as part of official outbreak investigations, followed by serovar Dublin accounting for 30%−40% of samples (15, 28). Nevertheless, there is some inherent bias in using veterinary diagnostic submissions to infer the prevalence of an organism on the broader population; therefore, these results need to be evaluated carefully.

**Table 1:** Estimation of *Salmonella* Dublin prevalence across different countries.

**Country (region)^a^**	**National control plan**	**Apparent prevalence**	**Method for prevalence estimation**	**References**
Canada (Ontario)	No	25%	Antibody testing on bulk tank milk and serum of 20 heifers in 100 herds	(10)
Denmark	Yes	9%	Bulk tank milk antibody testing every 3 months as part of national control program	(19)
Germany	No	0.7%	Isolation in samples of cecal contents on slaughterhouse (*n* = 283)	(20)
Great Britain	No	38%	Quarterly bulk tank milk antibody testing in 401 herds	(9)
Sweden	Yes	1%	Bulk tank antibody milk testing in 4,683 herds	(21)
The Netherlands	Yes	9%	Bulk tank milk antibody testing every 4 months as part of national control program	(22)
United States	No	0.7%	Bulk tank milk PCR in 234 herds	(23)
United States (New York State)	No	0.9%	Bulk tank milk antibody testing in 4,896 herds	(24)

a When no region is specified, the study was aimed at being representative of the whole country.

**Table 2:** Prevalence and antimicrobial susceptibility patterns of *Salmonella* Dublin in bovine isolates at the Michigan State University Veterinary Diagnostic Laboratory from 2018 to 2022.

	**Year**				
	**2018**	**2019**	**2020**	**2021**	**2022**
Number of *Salmonella* spp. isolations in bovine samples	206	202	223	186	131
Number (%) of *S*. Dublin isolations	26 (12.6%)	36 (17.8%)	36 (16.1%)	22 (11.8%)	25 (19.1%)
Antimicrobial susceptibility of *S*. Dublin isolates^a^					
Ampicillin	0%	0%	0%	0%	0%
Ceftiofur	4%	6%	8%	18%	12%
Clindamycin	0%	0%	0%	0%	0%
Danofloxacin	85%	80%	91%	90%	88%
Enrofloxacin	65%	83%	91%	90%	88%
Florfenicol	3%	0%	2%	9%	4%
Neomycin	0%	0%	0%	0%	0%
Penicillin	0%	0%	0%	0%	0%
Sulfadimethoxine	4%	0%	0%	4%	0%
Trimethoprim/Sulfamethoxazole	85%	100%	100%	95%	96%
Tetracycline	0%	0%	0%	4%	0%
Tulathromycin	62%	88%	66%	50%	68%

a Expressed as the percentage of susceptible isolates.

For determining the prevalence of *S*. Dublin, different approaches may be taken, depending if the focus is the herd or within-herd prevalence. The use of fecal culture or serology has different strengths and limitations when determining prevalence. This will be discussed in the diagnosis section. However, sampling must be performed serially to categorize an animal as a carrier, transiently infected, or negative (29). Similarly, herd sampling must occur over time, as different factors may affect the shedding of the bacteria in the infected animals (29). For example, the prevalence of *S*. Dublin in Danish herds varied between 8 to 76% when samples were measured every 3 months for 1 year from 2000 to 2002 (29). Studies where S. Dublin has not been isolated should be taken cautiously due to intermittent shedding or animal selection. For example, Cummings et al. (30) did not isolate *S*. Dublin during the years 2004–2005 in the northeastern US because the samples in the study were collected from animals with clinical digestive disease and not respiratory; therefore, cases might have been missed. In addition, studies including samples from clinically ill animals may overestimate regional or within-herd prevalence. Nonetheless, it is still a valuable approach to differentiate positive and negative herds. Serology has been widely used to determine prevalence in herds (31–33). However, the age of the animal and the type of sample used may affect the correct classification of herds and animals. For example, if only milk is submitted for testing, non-lactating animals may be missed, and the within-herd prevalence may be underestimated.

### 2.2 Human health hazard

*Salmonella* Dublin is a zoonotic bacterium that can cause rare but severe illness in humans, and it is characterized by acute gastroenteritis and bacteremia (5). The case fatality for *S*. Dublin has been reported as the highest compared to other *Salmonella enterica* serotypes and has been described as six times greater than *S*. Typhimurium (34). The consumption of raw milk or raw dairy products has been associated with outbreaks of human salmonellosis caused by serovar Dublin (7, 35–37). However, farm personnel, veterinarians, and any person in direct contact with cattle are at risk of infection by accidentally ingesting animal feces or fluids (38). Moreover, *S*. Dublin has been isolated from the hide of dairy cull cows in processing meat plants, where it can constitute a risk for carcass contamination and a human health hazard (39).

In the US, the Foodborne Disease Active Surveillance Network determined an increase in the incidence of human *S*. Dublin by 7.6 times from 1968 to 2013 (5). The same study determined an increase in hospitalization from 68 to 78%, and an increase in mortality from 2.7 to 4.2%, when comparing the periods 1996–2004 with 2005–2013 (5). In a case-control study performed in Denmark, human salmonellosis due to *S*. Dublin was associated with an adjusted mortality of 12.4%, which was 12 times greater than the mortality in the control group (40). In the UK, *S*. Dublin was associated with 2% of human salmonellosis between 1949 and 1951 (41). Furthermore, *Salmonella* Dublin can cause long-lasting disease: in 17.7% of the cases, the condition lasted over 14 days with a 3.9% of mortality, which was higher than any other *Salmonella* serotype (41). As discussed in Section 2.4., *S*. Dublin has been characterized as an MDR bacterium to common antibiotics used for the treatment of bacterial infections in humans and animals. Therefore, *S*. Dublin is a pathogen that can affect human health severely and compromise the medical treatment. For that reason, it is fundamental to prevent and reduce the risk of infection from cattle to farm workers, animal care takers, and from animal derived food to humans.

### 2.3 Pathogenesis and clinical signs in cattle

**Figure 1:**
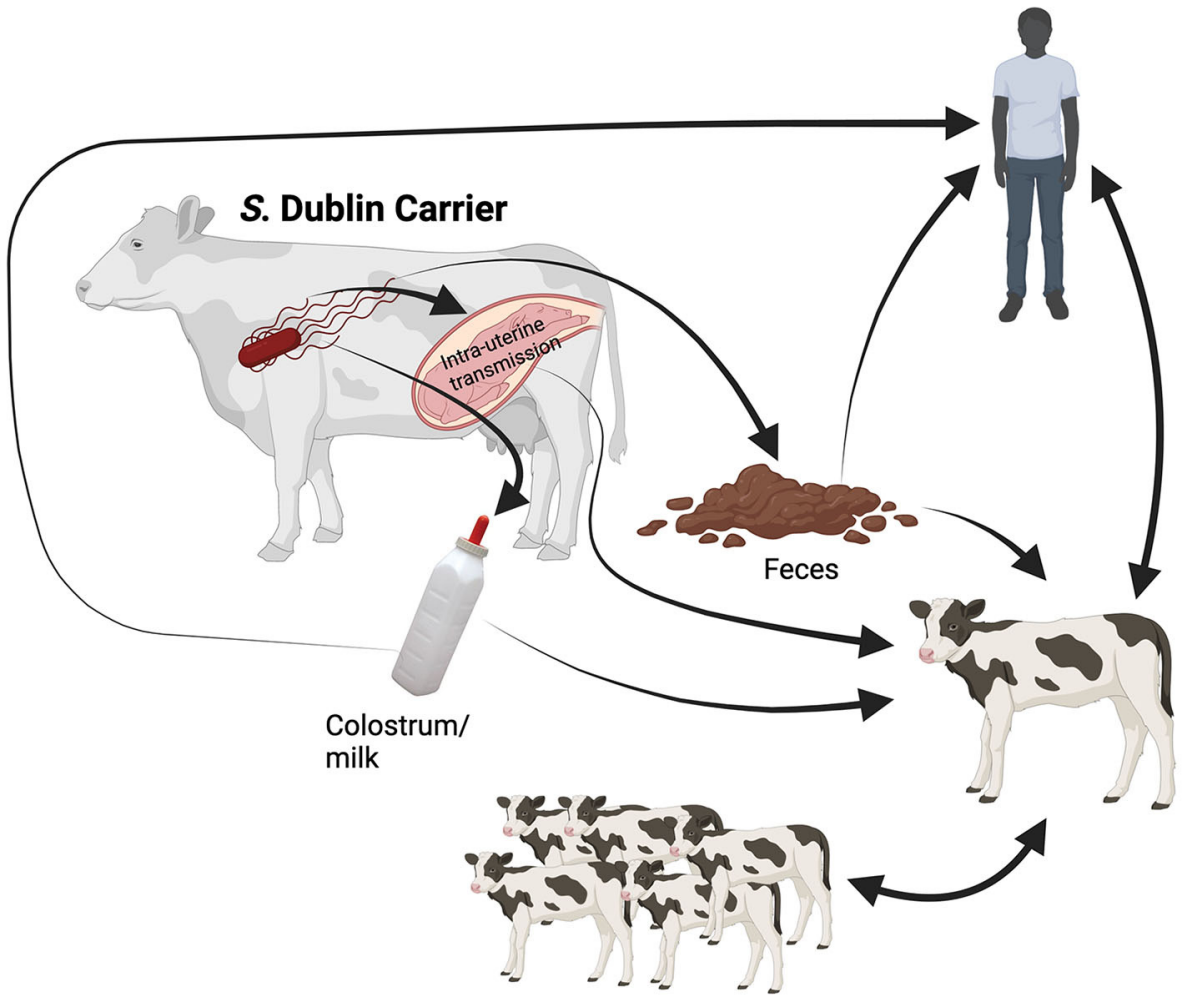
Illustration of the transmission routes for *Salmonella* Dublin. Symptomatic infected animals and latent carriers shed the bacterium to the environment under stress conditions, primarily in the peripartum. Once *S*. Dublin is shed in feces and secretions (saliva, colostrum, and milk) can survive in the environment. The newborn calf may uptake the bacterium via fecal-oral route at calving or by consumption of raw colostrum or milk from infected cows. Moreover, the infected calf will shed the bacterium to the environment, where susceptible calves will ingest *S*. Dublin through direct contact or fomite (contaminated surfaces or objects). In addition, the intrauterine infection of the fetus in the last trimester of gestation may occur, resulting in abortion or the birth of an infected calf. Finally, the zoonotic route will occur mainly in caretakers working with symptomatic animals and latent carriers at calving. The human will uptake *S*. Dublin from feces and secretions during calving assistance, cleaning equipment or facilities, manipulating raw colostrum and milk, or close contact with sick animals. Created with BioRender.com.

*Salmonella* Dublin is a rod-shaped, facultative anaerobic, Gram-negative bacterium host adapted to cattle. It can produce enteritis and septicemia in bovine and severe disease in humans (5). The routes of infection for *S*. Dublin are shown in Figure 1. Briefly, the most common route of infection for *S*. Dublin is oral (42). Susceptible animals may uptake this bacterium by ingesting feces or body fluids (milk, saliva, and nasal secretion) from infected animals or contaminated environments (2, 42). In addition, vertical transmission with abortion in the last trimester of gestation or the birth of a congenitally infected calf has been reported (4, 43–45). One study determined the presence of *S. enterica* in the newborn calf. The results suggested that *S. enterica* was isolated from 50% of the enrolled calves and from samples that included lymph nodes and intestinal tissue (45).

Additionally, experimental evidence suggests less common routes of infection through the upper respiratory tract (42) and mammary gland in cows under 60 days of lactation (46), although this study injected the bacteria directly into the udder in higher numbers which might not reflect natural exposure situations. In experimental conditions, it has been described that the minimum dose for infection that results in clinical disease and shedding of *S*. Dublin in animals younger than 6 months of life is 10^6^ colony-forming unit (CFU) (2). However, this dose might depend on factors such as the strain used for infection (2, 47). In addition, experimental infection of mature cows with the administration of 10^10^ and 10^11^ CFU oral or intravenously resulted in diverse clinical manifestations, ranging from no signs to severe disease and abortion, which might be dependent on the strain used in the studies (44, 48). The incubation period has been determined as 12–72 h (49). The infectious dose might have importance in individual susceptibility to the pathogen and in the subclinical presentation of this disease.

Once *S*. Dublin enters the animal, it colonizes the digestive tract invading and multiplying in the enterocytes. From the distal ileum, it is translocated by efferent lymphatics from the mesenteric lymph node (50). After the translocation, *Salmonella* can rapidly disseminate and produce systemic disease. Furthermore, *Salmonella* can survive as facultative intracellular bacteria in numerous organs (spleen, liver, and lungs) and lymph nodes, allowing them to elude the adaptive immune response (38). The adaptation of *S*. Dublin to cattle has been linked to the selection of variants that can evade the innate response in the host and reduce the inflammatory response in the intestine's mucosa, which facilitates the dissemination (51). This process has been achieved by mutation within the host, resulting in the acquisition of genetic elements that encode specific virulence factors or the loss of specific genes to survive the particular conditions in the host environment (51, 52). In contrast, the infection of other animal species is accidental (52).

Several virulence factors have been associated with *Salmonella*, such as *Salmonella* Pathogenicity Islands (SPI), toxins, flagella, fimbriae, and virulence plasmids (53). *Salmonella* Dublin encodes the Type III Secretion System from SPI-1 and SPI-2, which allows it to invade the intestine and spread to systemic sites, respectively (54). Additionally, *S*. Dublin encodes the Type VI Secretion System from SPI-6 and SPI-19, which allows the injection of effector proteins into cells, increases the virulence, and in experimental settings, contributes to interbacterial competition (55). In addition, *S*. Dublin has the pSDV plasmid with a spv operon that encodes a toxin associated with the host's cellular apoptosis (53). Furthermore, the plasmid contributes to increased virulence, the systemic presentation of the disease, and encoding of antimicrobial-resistant genes (47, 53). Moreover, *S*. Dublin expresses flagella encoded by the gene *fliC* (56). The flagella allow motility and, through chemotaxis, enables the bacterium to respond to changes in the host environment (56). *Salmonella* Dublin also has fimbriae that aid in the adhesion to the host cells and virulence plasmids (53). Finally, *S*. Dublin virulence factors had been associated with an enhanced intracellular proliferation in intestinal and extraintestinal tissues, leading to severe diarrhea and mortality in experimental conditions (57). However, virulence factors will influence the infection depending on the host's immunity, and immunity is crucial in the outcome of clinical salmonellosis, especially in young calves with an immature immune system (58).

Compared to other *Salmonella* serovars, the clinical manifestation of *S*. Dublin is more severe due to a more invasive capacity in cattle (59). However, the clinical signs related to *S*. Dublin infection in cattle will depend on the animal's age and the endemicity of the pathogen on the farm, as endemic farms will have persistent infected animals (29). The clinical manifestation of *S*. Dublin infection is most common in calves 2–12 weeks old (42, 60); although it can affect cattle of all ages. A peracute presentation may occur in calves with sudden death in 1–2 days due to endotoxic shock. This presentation is more common in naïve herds (2, 3). Even though *S*. Dublin is an intestinal bacterium, the most distinctive clinical signs in acute infections of calves are pneumonia, respiratory distress, and hyperthermia (3, 17). In addition, calves show signs of obtundation, lack of appetite, diarrhea, arthritis, and meningoencephalitis. Chronic infection is more common in calves 6–8 weeks old that survived an acute infection and is characterized by growth retardation, loose stool, and lameness due to arthritis (2). The morbidity in outbreaks of *S*. Dublin has been reported between 10.5 and 34.8%, mortality between 2.3 and 18.2%, and case fatality of 26.4% for dairy calves (3, 61). As a point of comparison, respiratory disease is a common cause of morbidity and mortality in pre-weaned calves. It has been reported that respiratory disease mortality ranges from 2.8 to 14% and a case fatality of 6.0% (62–64). Considering the information in published studies, *S*. Dublin exceeds the mortality and case fatality of the common calf respiratory disease.

In unexposed adult animals, *S*. Dublin may lead to fever, bloody diarrhea, and in extreme cases to death. However, signs might be unnoticed in herds with a history of *S*. Dublin; the typical presentation is a slight fever and mild diarrhea, which can be accompanied by a sudden reduction in milk production (2, 38). As previously mentioned, abortion in the last trimester of gestation is another clinical manifestation in mature cows due to bacteremia (4).

Nonetheless, *S*. Dublin infection may generate persistent infections without clinical signs of disease, except for a reduction in the milk yield (2). These animals have been called “latent carriers.” These carriers host the bacteria in lymphoid tissue and periodically shed the pathogen in feces or fluids when the immune system is compromised or challenged, as in calving or transportation (29, 43, 65). Therefore, latent carriers are a source of infection for the herd, and identifying and culling these animals is essential for controlling the spread of the disease within and between herds (66, 67). Factors associated with an animal becoming a latent carrier are described in the Section 3.

### 2.4 Antimicrobial resistance

The prevalence of MDR *S*. Dublin is associated with geographical location. For example, although *S*. Dublin is considered one of the most common MDR serotypes in the US (17), MDR is not a common phenomenon in the European *S*. Dublin isolates (68). However, *S*. Dublin MDR can reduce the success of treatments, delay recovery, and increase mortality and costs in humans and cattle (18). As mentioned in the previous section, the infection of dairy calves with *S*. Dublin may produce a severe disease that may require antibiotic treatment. However, if antimicrobial treatment is administered without testing for drug susceptibility, the animal will not only fail to recover, but this practice will contribute to enhancing pathogen resistance (6). Hence, this will have negative consequences for human health due to the zoonotic nature of this agent (6). Furthermore, there are limited antimicrobial options to treat animals with clinical signs effectively, and some of those drugs are not labeled for treating *S*. Dublin infections, as will be discussed later in this section.

In the US, a study performed between 1996 and 2013 found that *S*. Dublin had a 43% higher prevalence of MDR compared to other *Salmonella* isolates (5). The National Antimicrobial Resistance Monitoring System (NARMS) reported that among *S*. Dublin isolates, 84% were resistant to five or more classes of antimicrobial drugs, and 57% were resistant to seven or more antimicrobial classes (5). Furthermore, an increase from 29 to 79% was observed in the proportion of isolates resistant to one or more antimicrobial classes when comparing 1996–2004 with 2005–2013 (5). Studies including samples from cattle, retail meat, and humans have reported *S*. Dublin antimicrobial resistance (AMR) to ampicillin, chloramphenicol, neomycin, tetracycline, streptomycin, sulfonamide, amoxicillin/clavulanic acid, and ceftriaxone (5, 6, 13, 17, 69). A similar situation has been described in other countries; in China, bovine isolates had AMR between 92 and 98% for tetracycline, sulfamethoxazole, and ampicillin (70). In the same study, human isolates were resistant to ceftiofur, chloramphenicol, tetracycline, and sulfamethoxazole in 40%, 50%, 50%, and 70% (70). Furthermore, in Canada, a study determined a high resistance to streptomycin, β-lactams, gentamicin, chloramphenicol, sulfisoxazole, and tetracycline from bovine samples (71). In Germany and the UK, *S*. Dublin has not been associated with significant AMR in bovine samples collected from cattle (68, 72) or beef products (73). This might be explained by a difference in the expression of plasmids and genes associated with AMR and MDR between regions, as specific AMR plasmids have been reported in the US and China but not in Europe (68).

In the US, it was reported that *S*. Dublin is susceptible to gentamicin, amikacin, cefoxitin, cephalothin, enrofloxacin, meropenem, and azithromycin (6, 17). Even though this pathogen is susceptible to enrofloxacin, this drug is only allowed to treat specific bovine respiratory disease pathogens in non-lactating cows and dairy replacements younger than 20 months. Hence, enrofloxacin is not labeled as a treatment for *S*. Dublin infections, and the extra-label use of this drug is prohibited for food animals in the US. Although most producers and veterinarians would treat respiratory disease without a pathogen isolation diagnosis, current US regulations imply that enrofloxacin cannot be used when *S*. Dublin is suspected or confirmed. This complicates the proper treatment of sick calves and potentially might increase the use of drugs to which *S*. Dublin has reduced susceptibility. The antimicrobial susceptibility pattern of *S*. Dublin isolated has largely remained unchanged in recent years (Table 2), with *S*. Dublin being generally susceptible to only four antimicrobials. Among those four, only Trimethoprim/Sulfamethoxazole has been labeled for treating *Salmonella* infections.

Different mechanisms have been described for the observed antimicrobial resistance, such as the production of enzymes that degrade or produce structural changes in antimicrobial agents, membrane impermeability, activation of antimicrobial efflux pumps, modification of cellular target for antibiotics, and biofilm formation (74, 75). Additionally, *S*. Dublin MDR has been linked to plasmid-borne resistant genes (6, 70, 75–77). Through whole genome sequencing, Srednik et al. (6) determined that the most common genes associated with AMR in the US were *sulf2, tetA, aph(3*″*)-Ib, floR*, and *bla*_CMY−2_. In China, similar genes were associated with AMR, and in a lower proportion *aph(3*′*)-Ia* and *bla*_TEM−1B_ (70). In Canada, the AMR was associated with *str*_AB_*, bla*_CMY−2_*/bla*_TEM−1_, *aadB, floR/cmlA, sul2*, and a class A *tetA* (71). The mentioned genes conferred resistance to sulfonamide, tetracycline, aminoglycosides, chloramphenicol, and beta-lactams. Furthermore, in the mentioned studies, the MDR plasmid, IncA/C2, was present in 81.8% and 98.6% of all bovine isolates (6, 70). Hence, IncA/C2 might represent a critical MDR plasmid and a distinctive feature from bovine isolates with AMR (68, 70).

### 2.5 Economic impact

To date, a formal assessment of the financial impact of *S*. Dublin in the US has not been conducted. However, the economic importance of this pathogen in dairy farms is associated with the treatment of sick animals, reduction in milk yield, abortion, culled animals, mortality, cost of disinfection of facilities, and increased working hours during outbreaks (78, 79). Additionally, latent carriers contribute to the endemicity of the pathogen in the herd, spreading the pathogen and infecting susceptible animals, increasing the risk of outbreaks and associated costs. In Denmark, Nielsen et al. (78) utilized a simulation model to estimate the economic effects of *S*. Dublin for 10 years in a herd. The model included 12 scenarios based on three herd sizes and four management levels. The results indicated that the more significant economic losses occurred in the first year after the introduction of *S*. Dublin. The losses increased as the herd size increased, and the management practices decreased. From this analysis, milk yield losses in active and latent carriers were the most impactful. Even though these scenarios were adapted to dairy herds in Denmark, and some information might differ from other dairy systems, it provides an estimation of the economic implication of this pathogen. For example, in a 200 head herd with poor management practices, losses were estimated at 326 EUR per stall in the first year and 188 EUR per stall as an annual average of 10 years (78). Furthermore, this simulation reinforces the impact of basic management practices on the herd's economy. Those practices will be discussed in the prevention and control section.

## 3 Risk factors for infection

The identification of risk factors for infection with *S*. Dublin in animals and herds has come from epidemiological studies primarily conducted in Europe. These studies have reported factors associated with the host, farm practices/environment, and the agent. A summary list of risk factors can be found in Table 3.

**Table 3:** Summary table for risk factors associated with infection and shedding of *Salmonella* Dublin and preventative strategies for dairy herds.

**Risk factor**	**Prevention**	**References**
Poor hygiene (maternity barn, pre-weaned area, tools used to feed or treat sick animals)	Frequent sanitation of barns and water troughs, avoid high-pressure washing	(38, 66, 67, 80–82)
Purchase of non-tested livestock or from positive facilities	Purchase of cattle tested for *S*. Dublin	(31, 83–86)
Age (animals younger than 12 weeks, heifers between 12 months and calving)	Avoid contact between different age animals, culling latent carriers	(29, 42, 60)
Stressors (calving, transportation)	Reduce stress and frequent sanitation of barns and trailers used for high-risk groups	(29, 38, 87–89)
Delayed separation of the newborn from the dam in endemic herds	Prompt separation of the newborn. Identification and culling of latent carriers	(66, 67, 81, 90, 91)
Feeding pooled colostrum, raw colostrum, or waste raw milk to calves	Pasteurization of colostrum and milk	(38, 81, 92)
Contact of young stock with older or adult cattle	Strict age group housing and physical separation of different groups	(81)
Use of maternity as a recovery pen	Avoid housing sick animals in maternity pens	(93)
Overstocking	Correct animal density by pen size	(67, 94)
Sick and healthy calves in the same area	Isolation of clinically ill animals, disinfection of tools and housing	(60, 81, 90)
Close contact with neighboring animals from positive herds	Limit access to neighboring operations	(31, 32, 86)
Presence of rodents and no use of personal protective equipment by veterinarians and visitors	Strict internal biosecurity	(22, 95)

### 3.1 Host factors

The animal's immune status has a fundamental role in the control of new infections. Calves are born with an immature immune system that makes them highly susceptible to diseases early in life (96). In endemic herds, therefore, newborn calves exposed to contaminated environments or infected cows are at high risk of uptake *S*. Dublin by oral ingestion of feces, colostrum, or other body fluids and developing clinical disease (38). A similar situation is described for pre-weaned calves, as components of the adaptive immune system start to mature progressively from the second week of life (96); therefore, contact with *S*. Dublin in the environment or feed may lead to infection (38).

Moreover, animals experiencing concomitant diseases are at greater risk of infection or shedding of *Salmonella* spp. (97). In mature cows, liver fluke has been associated with an increase of over 14 times in the likelihood of *S*. Dublin infection compared to healthy cows (83). Likewise, concurrent infection with Bovine Viral Diarrhea Virus, an immunosuppressant virus, has been associated with a more severe clinical presentation of *S*. Dublin in calves (98).

### 3.2 Latent carrier factors

The importance of latent carriers in the epidemiology of *S*. Dublin is critical as they are defined as animals with a persistent infection without clinical signs but with an intermittent shedding of the pathogen to the environment through feces or secretions (2). However, latent carriers may be clinically ill or reactivate the shedding when the immune system is compromised (2). There is an association between age and the productive stage of the cattle with the risk of new infections and latent carrier status. For example, calves younger than 12 weeks of age are highly susceptible to infection of *S*. Dublin (42, 60). Repeated infection in young calves leads to a high proportion of resistant adult cattle and an increase in abortions in the last third of gestation (80). Moreover, it was reported that heifers infected between 1 year of life and calving were 11 times more likely to become a latent carrier than cows infected in mid or late lactation (29). A similar situation was described for cows infected in the peripartum, as they were 4 times more likely to become latent carriers (29).

Additionally, stress is an important factor associated with the infection of susceptible animals and the shedding of *S*. Dublin in latent carriers (29). Calving and transportation have been identified as periods of increased stress and shedding of *S*. Dublin (29, 87–89). Furthermore, any management practice that may increase the stress of the animals and the direct contact with infected animals that are actively shedding *S*. Dublin may result in infection of susceptible animals or outbreaks of disease (38).

### 3.3 Management practices and environmental factors

Different management strategies increase the prevalence of the herd and the susceptibility to infection for calves and cows. One of the most important risk factors for introducing and spreading infection into a susceptible herd is the purchase of livestock from facilities positive to *S*. Dublin or the purchase of non-tested animals (31, 83–85). The purchase from test-positive facilities has been associated with an increase between 2.5 and 4.2 times in the likelihood of infection of the new herd compared to animals purchased from negative facilities (31, 83). Furthermore, comparing the purchase of 1–10 animals with the purchase of more than 20 animals from test-positive herds, the likelihood of herd infection increased from 3.8 to 7.4 (86). The basic reproduction ratio (R0) for *S*. Dublin has been estimated at 1.1–2.7, meaning that 1–2 susceptible animals will be infected by introducing an infectious cow (84, 99). However, the screening and diagnosis of infected animals and latent carriers before purchase may be complex, as discussed in the diagnosis section.

In addition, poor hygiene has been identified as a highly influential risk factor, especially in maternity and calving pens (80). As mentioned previously, the shedding of *S*. Dublin increases around calving, and there is a greater probability of contact between the pathogen, newborn calves, and susceptible cows. Poor hygiene has been defined as visible or buildup of manure in the environment, the legs and udder of the cows, wet bedding, and infrequent manure removal (80). In fact, farms that added bedding material to calving areas once or twice per week had lower odds of being *S*. Dublin positive compared with farms that added bedding less than once weekly (10). A study comparing different levels of hygiene through a simulation model, determined that in a herd of 200 cows the probability of *S*. Dublin spread was 77 vs. 92% when comparing excellent with poor hygiene (80). Management practices associated with increased risk of *Salmonella* spp. infection of newborn calves are pooling and feeding raw colostrum in endemic herds (38, 92), delayed separation of the newborn calf from the dam (81), overstocking of the maternity pen (67, 94), increased number of cows calving outside the maternity area, and the use of the maternity as recovery pen (93). All these managements increase the exposure of newborn calves to feces and body fluids that might be contaminated with *S*. Dublin. In calf facilities, overstocking, poor nutrition, different age groups in a pen, and poor hygiene might lead to a fast spread of diseases. Additionally, clinically ill calves that are not isolated represent a hazard for pen mates or closely housed calves, as they are active shedders (38, 60, 81, 90). In endemic herds, feeding waste raw milk or the provision of contaminated water or feed has been associated with a greater probability of infection (38, 81).

There is no agreement in published studies if herd size is a risk factor for *S*. Dublin infection and spread. Several studies have determined that larger herds have a greater herd susceptibility (31, 83, 85, 86). However, other studies have not found herd size influential in infection, shedding, or spread of *Salmonella* (93, 100). Despite herd size, the lack of personal protective equipment may significantly increase the risk of introduction and infection of herds when veterinarians and visitors enter a facility (22). In addition, Tablante and Lane (95) suggested that wild mice could act as a reservoir and potentially transmit the pathogen to cattle or allow the persistence of the pathogen in the environment.

Concerning the environment, the prevalence of *S*. Dublin in nearby dairy facilities was associated with a greater risk of introduction into the herd (31, 32, 86). For example, a dairy farm with two or more positive neighboring facilities in a radius of 2 km is 1.7 times more likely to be infected than farms with no positive neighbors (86). This information may be critical in systems where cows have access to pasture and the possibility of close contact with neighboring herds exists. In systems where cows are housed in barns with no pasture access and higher biosecurity, this factor might be less influential in introducing *S*. Dublin. However, local herd prevalence was associated with 1.8 times greater risk of *S*. Dublin introduction (31). In addition, surface water contaminated with *S*. Dublin can lead to a fast spread of infection in animals with access to ponds, lakes, or rivers (83). Lactating and dry cows were 2.1 times more likely to shed *Salmonella* when having access to surface water than cows with no access (100). Finally, in the US, a seasonal association has been described; cows have a greater likelihood to test positive in summer, spring, and fall when compared to winter months (100). However, the authors speculate that this difference may be due to the intensity of the sampling in comparison to other studies, and the states included in the study had relatively similar weather conditions.

### 3.4 Agent factors

The main virulence factors associated to *S*. Dublin in cattle were presented in Section 2.3. In addition, *S*. Dublin is able to cross the placenta and infect the fetus. Therefore, maintaining latent carriers in the herd may contribute to the prenatal infection of newborn calves and keep the infection in the herd (4, 43–45). Hanson et al. (45) performed a study and found that 50% of the newborn calves enrolled were positive to *S. enterica* immediately after birth and before consuming colostrum. However, neither the dams nor the calves were tested to determine the serotype, and the sample size was small.

In addition, *S*. Dublin can survive in the environment, increasing the risk of infection of susceptible animals (101). Dairy cows in intense grazing herds had 13.2 times greater odds of infection with *S*. Dublin than cows from less intense grazing systems (83). This increase was explained by a greater fecal contamination of the pasture and a reduction in the time between manure spread and grazing. Likewise, a greater risk for shedding of *S*. Dublin has been identified in cows that consumed roughage from fields where manure was applied in solid or liquid form but not plowed in the same season (100).

## 4 Diagnosis

There are two main approaches for the diagnosis of *S*. Dublin: the detection of bacteria and the detection of antibodies. Both methods have advantages and limitations, and they can be performed in individual animals or the herd. This review will provide a basic approach to diagnosis, as techniques have been described in depth elsewhere (2).

### 4.1 Detection of bacteria

Bacteriological culture has been useful for isolating and identifying *S*. Dublin to trace infections and active shedders (2, 102). Bacteriological culture can be performed utilizing a variety of samples, including feces and fluids from live animals, organs from necropsies, aborted fetuses, or environmental samples. This method aims to isolate live bacteria (2). Thus, the procedure involves a pre-enrichment and a selective enrichment to allow bacterial growth, followed by plating and confirmation (2). This method has been described as more relevant in acute infections and clinically ill animals, as the correct isolation will depend on the number of bacteria in the sample (2, 102, 103). For that reason, the sensitivity of this assay has been described as low (104), and it has a limitation that latent carriers might be undetected due to the intermittent fecal shedding of *S*. Dublin. Bacteriological culture using samples from manure pits, drinking water, milk filters, and feces of clinically ill animals was associated with a sensitivity of 45%, 5%, 7%, and 38% for detecting *S*. Dublin, respectively (105). In post-mortem examination of clinically ill animals, the collection of tissues from the lungs, spleen, liver, intestine loops, gallbladder, intestinal content, and lymph nodes increases the probability of bacteria isolation (3, 106). A potentially more sensitive and faster method for the detection of genetic material of *Salmonella* is the polymerase chain reaction test (PCR) or real-time PCR (107). Persson et al. (108) described an *S*. Dublin-specific real-time PCR. The procedure for this method requires a pre-enrichment of the sample from lysates or extracted DNA (107). To increase sensitivity, a DNA extraction is recommended (107). However, the specificity of the assay in comparison to the numerous other *Salmonella* serotypes is yet to be determined. Currently, the most commonly utilized PCR assays do not allow serotype determination, and bacterial culture is recommended after performing a PCR (2). There is evidence of a multiplex PCR to discriminate *Salmonella* Enteritidis, *Salmonella* Pollorum, and *S*. Dublin (109). This assay is based on detecting three genes (*tcpS, lygD*, and *flhB*), where *tcpS* exists only in the serovars mentioned (109).

### 4.2 Detection of antibodies

The detection of immunoglobulins against *S*. Dublin is performed through an Enzyme-linked immunosorbent assay (ELISA). This method has a lower cost than bacteriological culture, and it can be used as a monitoring strategy in the herd to identify latent carriers during programs of control and eradication (67, 110). *Salmonella* Dublin is part of the D-serogroup of *Salmonella* and has the antigenic factors O1, O9, and O12; therefore, cross-reaction between serovars sharing O antigens may occur (111). The ELISA is based on detecting immunoglobulins directed to the LPS O- antigen from serum, milk, and bulk tank milk (BTM) samples (112, 113). The kit is commercially available in several countries for monitoring and surveillance of *Salmonella* infections in cattle herds (Applied Biosystems™, Massachusetts, USA). The results provided in this ELISA are semi-quantitative for antibody concentration as they are expressed in ODC% (optical density coefficient). The interpretation of the result is based on an estimated cut-off point to determine positive animals depending on the sample. The ODC% cut-off for serum, milk from an individual, or BTM is 35 ODC%. A positive correlation exists between the ODC% and antibody concentration in a sample. In BTM, the greater the ODC%, the higher the spread of infection in the herd (32). To identify latent carriers and the intermittent shedding of *S*. Dublin, sequential samples should be obtained from individual animals by using milk or serum samples. For example, studies from countries with eradication plans recommend that cows be sampled quarterly (31, 32). The limitations of this assay include that the sensitivity and specificity are age-dependent, as it performs better as a diagnostic test in animals older than 100 days (104). Additionally, milk samples have the limitation that only lactating cows can be tested (2, 105). Furthermore, when BTM samples are used, there is the probability of diluting immunoglobulins in cows' milk with low or no titers (110). Even though the aim of using BTM samples is to estimate herd prevalence, it is still unclear what is the limit of detection of the assay in BTM samples (i.e., the lowest proportion of positive animals needed for the sample to be correctly classified as positive). However, simulation models suggest that the apparent herd prevalence would need to be between 0.3 and 0.55 for a bulk tank milk sample to be declared positive with a 35 ODC% (110).

### 4.3 Postmortem examination

There are no pathognomonic lesions in internal organs for infections with *S*. Dublin. However, while considering the age of the animal and the clinical signs, necropsy may be helpful to guide diagnosis or for sample collection. In calves with clinical presentation, the gross pathologic findings in the lungs include pulmonary congestion, suppurative pneumonia, and chronic bronchopneumonia, depending on the severity of the clinical case (17, 106). The intestinal lesions may include diffuse catarrhal hemorrhagic enteritis, ileitis, and mesenteric lymphadenitis (3, 106). The intestinal content is watery, malodorous, and may contain mucous, blood, or fibrin clots (3, 106). Moreover, the liver is enlarged with rounded edges and hemorrhagic areas on the capsular surface, and gelatinous edema of the gallbladder (3). Jaundice and splenomegaly are also common postmorten findings in cases of *S*. Dublin (114). In some cases, swollen joints may be a finding (17).

## 5 Treatment

The treatment of *S. enterica* infection is directed to provide supportive therapy by correcting the electrolyte imbalance, dehydration, and management of inflammation (106, 115). However, salmonellosis due to serovar Dublin may be difficult to treat and fatal. Electrolyte therapy can be provided orally or intravenously, depending on the calf's degree of dehydration and suckle reflex. Calves with strong suckle reflex and dehydration below 8% can be offered oral electrolytes, while calves with over 8% dehydration should be administered intravenous fluid (116). Calves experiencing systemic infection should be administered non-steroidal anti-inflammatory drugs to manage inflammation. Antimicrobial use to treat the clinical presentation of *S*. Dublin is controversial, as discussed in the section on antimicrobial resistance. Ideally, the appropriate selection of antimicrobials should be based on susceptibility testing (106). However, this might not always be an option in the face of an outbreak. Moreover, the nutrition of the sick calf is important for recovery as it is the source of calories. Milk can be offered in smaller portions throughout the day to encourage milk consumption (117). Additionally, the calf's environment should be clean with dry bedding, have access to water, and be protected from adverse weather conditions.

## 6 Management strategies for prevention and control

The strategies for prevention and control of *S*. Dublin should focus on reducing disease transmission by changing management practices and restrictions in the purchase of animals, detecting and managing infectious animals, and continued surveillance (81). This section will discuss the most critical management for preventing and controlling *S*. Dublin on dairy farms. Table 3 lists strategies to reduce the risk of infection and shedding of *S*. Dublin.

### 6.1 Sanitation

Research has demonstrated that practices associated with the cleaning and disinfection of the environment are key elements in the prevention and control of *S*. Dublin (66, 67, 81). The first step in decontaminating the environment and equipment is removing organic material (i.e., food, manure, bedding), as this can inactivate disinfectants. Secondly, it is necessary to rinse the surfaces with water and apply a detergent in all the areas to clean. A thorough rinse with water should follow this step. High-pressure washing should be avoided, especially in indoor housing, as it may spread contaminants and aerosols to the environment (81). The final step is the application of the disinfectant in the concentration and contact time described on the label, as any variation in the use of a disinfectant may affect its effectiveness. *Salmonella* is susceptible to most disinfectants if steps one and two have been appropriately performed (118).

Besides cleaning and disinfection, improvement in management practices, such as cleaning water troughs with chlorinated disinfectant twice a week, replacing bedding weekly, and not recycling the water used for flushing pens, have been associated with a decrease in *Salmonella* incidence in a herd experiencing an outbreak (66). Furthermore, *S*. Dublin in endemic herds from Denmark has been controlled by daily removal of manure, the cleaning and disinfection of the calving area at least twice a month, and new bedding added weekly (67). Although these practices may be unpractical in some herds, the sanitation of calving areas should be performed according to their use. Moreover, trailers used for the transport of animals should be cleaned and disinfected consistently (38). In the case of calves, it has been recommended that after disinfection, pens should be kept empty for a minimum of 2 days before new animals are housed in them (81). Furthermore, cleaning and disinfection should be a priority for all equipment used to manage sick calves or to feed calves, including tools used to harvest, store, and provide colostrum and milk, such as esophageal tubes, nipples, bottles, and buckets (38, 66). In addition, buckets used to feed water and starter in pre-weaned calves should be cleaned daily and positioned to reduce the risk of fecal contamination (106).

Even though feeding waste milk can be cost-effective, the provision of raw milk may increase calf morbidity and mortality due to the ingestion of pathogens (82). Therefore, another hygiene strategy to prevent and control *Salmonella* infection in pre-weaned calves is the pasteurization of colostrum and milk as it has been shown to reduce microbial populations including *S. enterica* species (92). However, adequate maintenance of the equipment and regular sampling of colostrum and milk is highly recommended as a tool to monitor the correct functioning of the pasteurizer (38, 82).

### 6.2 Stocking density and isolation of sick animals

Maintaining an adequate stocking density in mature cow pens (i.e., close-up, maternity, fresh cow), heifer pens, and pre-weaned calves housed in groups is a practice that can reduce the contact between animals, the contamination of the environment, and new infections. As mentioned in the latent carrier factors section, it is necessary to consider the existence of groups of animals that are more susceptible to new infections or to shed *S*. Dublin. For instance, in those groups, it is critical not to overcrowd. In addition, the calving area should not be used to house sick animals due to the risk of environmental contamination and infection of newborn calves (67). Moreover, young calves should not have access to or contact with older animals; therefore, strict age group housing in conjunction with adequate stocking density has been recommended to prevent and control *S*. Dublin (81). Finally, animals exhibiting clinical signs of *S*. Dublin infection should be isolated, and strict cleaning procedures should be in place (38).

### 6.3 Newborn management

The management of the newborn and the calving area is critical to prevent *S*. Dublin infections. Some considerations must be taken as latent carriers can reactivate the shedding of bacteria around calving (29, 89). Newborn calves should be separated from the dam as soon as possible after birth to avoid oral infection due to consumption of colostrum or feces from the dam or other adult cows. The correct management of colostrum is fundamental to preventing infectious diseases in young calves (119). The newborn calf should receive the first colostrum feeding within 4 h after birth (119). Ideally, calves in endemic herds should be provided with pasteurized colostrum. Moreover, having fewer personnel in charge of calving and colostrum handling was associated with preventing *S*. Dublin infections (67). However, this might be challenging and not be suitable for all farms. Thus, it is fundamental to provide training to the personnel to keep farm practices according to the established protocols.

### 6.4 Vaccination

Commercial and autologous vaccines have been used to control *S*. Dublin in herds. However, autologous vaccines have not been evaluated in published studies for their efficacy in preventing and reducing the clinical signs or the shedding of *S*. Dublin in dairy animals. A commercially available modified-live vaccine available in various countries (EnterVene-D, Boehringer Ingelheim) is recommended for animals older than 2 weeks with a booster after 12–16 days. The benefits of an attenuated-live *S*. Dublin vaccine are associated with a robust response at mucosal level due to its action on lymphoid tissue in the gut, and a robust cell-mediated immune response due to intracellular proliferation (120, 121).

The age for the first dose can be too late as calves may get infected with *S*. Dublin at birth or in the first hours of life. Moreover, limited research addresses the dam vaccination as an approach for producing antibodies that can be delivered to the newborn calf through colostrum (122). The evidence suggests that specific antibodies for *S*. Dublin are in a higher concentration in the colostrum of cows vaccinated 30 days before dry-off than in non-vaccinated cows (122). However, it remains unknown if those antibodies have a protective effect on the newborn calf. In addition, research evaluating intranasal and oral vaccination of 4-day-old calves suggests that those are safe routes (123, 124). Using these extra-label routes of administration reduced the disease severity as calves administered the vaccine had a reduced mortality rate compared to unvaccinated calves (124). However, the incidence of pneumonia, abnormal fecal scores, and the fecal shedding of *S*. Dublin were not reduced (123, 124). Furthermore, no differences were observed in the average daily gain or antibody concentration at 10 weeks and 10 months of life compared to control calves (124).

Additionally, few studies assessed the cross-protection between *S. enterica* with modified-live vaccines. Mohler et al. (106) found that calves younger than 2 weeks of life orally vaccinated with modified-live *S*. Typhimurium had less severe clinical signs, improved appetite, and reduced fecal shedding when challenged with *S*. Dublin compared to control calves. However, calves in that study were challenged with a dose of S. Dublin to induce disease and minimize mortality, and respiratory clinical signs were not assessed. Similar results were found using an attenuated-live *S*. Typhimurium vaccine on diarrhea and shedding of *Salmonella* Newport and *Salmonella* Cerro (120). Moreover, there is a study assessing the vaccination of the dry cow with an *S*. Newport bacterin to provide cross-protection in an *S*. Typhimurium challenge in calves fed colostrum from vaccinated dams. Despite higher serological titers, no difference in mortality, clinical signs, hematology, and fecal cultures were observed in calves fed colostrum from vaccinated cows and the control group (125). Based on this research, the cross-protection between *Salmonella* spp. and potential protection against *S*. Dublin in dairy herds is still in development.

### 6.5 Farm biosecurity

Although the *S*. Dublin status of the herd is not frequently known in countries without a control program, several European studies have found that avoiding the purchase of cattle from test-positive herds or herds with unknown infection status should be a consistent practice in dairy facilities to prevent the introduction of *S*. Dublin in the herd (31, 67, 83, 86). Similarly, pasture-based operations should prevent close contact with cattle from neighboring farms, as the introduction of *S*. Dublin can occur through direct contact (22, 32, 83, 86). The vertical transmission of *S*. Dublin can perpetuate the disease even in closed herds (43, 45, 83). Therefore, culling of latent carriers should be considered for control or eradication of *S*. Dublin (66, 67). However, this strategy might not be suitable for all herds as seroprevalence within the herd may range from 3 to 70% (105, 126, 127). Additionally, persistently infected herds may have a high percentage of seroprevalence for *S*. Dublin without clinical signs or frequent outbreaks (127). Therefore, producers might find it economically counterproductive to cull valuable animals.

For surveillance, the collection of serial samples from the BTM is an easier, non-invasive, and less expensive method to determine the status of a herd concerning the presence or absence of *S*. Dublin antibodies (33, 91). In addition, for control of *S*. Dublin in a herd, it is helpful to collect serial samples from individual animals to diagnose the pathogen in an outbreak or identify latent carriers, this can be done with individual ELISA o bacteriological culture (90, 91). Additionally, the proper use of personal protective equipment for visitors and veterinarians, the control of rodents, along with the prevention of cross-contamination by not using the same equipment for different tasks are encouraged (22, 38, 95). As part of the eradication plan in Denmark, herds positive for *S*. Dublin had to maintain a high level of internal and external biosecurity for an average of 3 years to become test negative and prevent the recurrence of infection (31). Therefore, consistency in management practices is critical throughout the time to control *S*. Dublin.

### 6.6 Farm personnel

Training should be provided to personnel working with animals regarding the risk of zoonotic diseases and their prevention. Particular attention should be put on personnel handling animals during an outbreak of *S*. Dublin or personnel handling animals in periods of stress, when latent carriers may reactivate the shedding of *S*. Dublin. While working with animals or cleaning equipment, personal protective equipment (coveralls, washable boots, gloves, masks, and goggles) should be used. In addition, personnel should remove personal protective equipment before leaving the farm, and boots should be cleaned and disinfected. During an outbreak of salmonellosis or in eradication programs, it is recommended to have personnel working exclusively with clinically ill animals or isolated carriers to avoid potential spread and cross-contamination to other animals or areas. Finally, it is crucial to consider the mental and moral cost of an outbreak of *S. Dublin* on animal handlers, as it is a disease that might result in high morbidity and mortality, extended working hours, and depletion of morale due to low treatment success.

### 6.7 Gene therapy and gene editing

Recent research has evaluated the possibility to use gene therapy or gene editing to inhibit virulence gene expression in *Salmonella* spp. (54, 128). The use of certain lactic acid bacteria (LAB) combinations and their degradation products has been associated with the downregulation of virulence genes in different pathogens associated with neonatal calf diarrhea, including *S*. Dublin (54). Specifically for *S*. Dublin, a combination of 61 LAB strains was able to downregulate the expression of virulence factor *fliC*, which was assessed with RT-qPCR (54). Additionally, current research explores using CRISPR/Cas9 to delete the plasmid-based *SpvB* gene by using a modified pCas9 plasmid in pathogenic strains of *S*. Gallinarum (128). The results are promising, as the manipulated strain did not induce clinical disease or gross pathological lesions in broiler chickens 36 days after the challenge (128). Even though there are no current reports on *S*. Dublin, this procedure might be applied to produce nonvirulent strains that could be used in vaccines.

### 6.8 Next-generation sequencing

Whole genome sequencing (WGS) has become a tool to investigate the epidemiology of diseases, and *S*. Dublin has not been the exception. This methodology makes it possible to compare the whole genome of pathogens, which is a tremendous contribution to understanding disease dynamics, and it is a tool that can be used for the surveillance and control of diseases (129). WGS has been used to characterize the proximity in *S*. Dublin clades and differentiate the AMR and MDR genes between different regions and continents (68, 130). In addition, it has been used to determine the clonal relationship of *S*. Dublin strains in cattle and food animal products, with the potential to track zoonotic outbreaks (7, 73, 131). Furthermore, it has been used to study the proximity between cattle and human strains of *S*. Dublin, their virulence, and AMR genes (70). WGS has been and will be critical to understanding the adaptation of *S*. Dublin in different areas and the challenges associated with specific gene expression (68).

## 7 Future work

There is significant research related to *S*. Dublin in dairy cattle and operations. However, some areas remain less explored. Estimates of regional and national prevalence in many dairy production areas worldwide have yet to be determined. This type of research is associated with high costs, extended periods of sampling, and regional variation that might make the comparison difficult. Even though there are published studies, these have included specific regions, and in some cases, samples have not originated only from dairy farms. In addition, some studies have focused on determining several serovars of *S. enterica*; therefore, the study designs have not been specifically developed for *S*. Dublin. Moreover, studies determining the prevalence of *S*. Dublin have been performed mainly with samples submitted to VDLs, which might bias prevalence estimation (11, 12). Without knowing the local situation concerning *S*. Dublin, it is difficult to understand the real impact of this pathogen on cattle health and establish appropriate prevention strategies. Whole genome sequencing is becoming a tool of increasing importance in epidemiological studies, and it is an adequate method to investigate outbreaks, virulence factor, and their similarities between different regions and continents.

Currently, there are no published studies assessing the economic impact of *S*. Dublin in other production systems beyond Denmark. Worldwide, few studies have addressed the financial losses related to *S*. Dublin using simulation models (78, 79). However, the results of these studies might not be extrapolated to productive systems different from those described in the models. The simulation models might be an approach to estimating the economics of *S*. Dublin in a herd.

Another area with growing but limited information is gene editing or gene downregulation to prevent the severity of *S*. Dublin infections. Also, there is limited information is the use of vaccines to prevent *S*. Dublin in calves. As mentioned in the vaccination section, few studies have evaluated the effectiveness of the passive immunity provided to newborn calves by vaccinating the dam prepartum. Moreover, no studies evaluated the effect of vaccinating pregnant latent carriers on the disease transmission and severity in the offspring. Finally, few studies have addressed the effectiveness of extra-labeled routes of administration of vaccines against *S*. Dublin administered to young calves in field trials. Currently, no studies have addressed an integrated approach to prevent and reduce the devastating consequences of *S*. Dublin in neonatal and pre-weaned calves.

## 8 Conclusions

*S*. Dublin severely affects cattle and human health. Recent reports indicate that its prevalence has increased in several countries in the last several years, making it an emergent pathogen. Information is available on pathogenicity, antimicrobial resistance, risk factors, and preventive management practices. However, more research is still needed on the economic impact of outbreaks or endemic disease on herds and the effectiveness of strategies that could be implemented in dairy facilities to prevent and control *S*. Dublin.

## Author contributions

AV-M: Conceptualization, Formal analysis, Methodology, Writing—original draft. RC-V: Methodology, Writing—review & editing. FC-N: Supervision, Writing—review & editing. RM: Resources, Writing—review & editing. AA: Conceptualization, Funding acquisition, Methodology, Supervision, Visualization, Writing—review & editing.
